# Supplementary material for: Microbial Contamination of Photographic and Cinematographic Materials in Archival Funds in the Czech Republic
Source: Microorganisms. 2022 Jan 12;10(1):155. doi: 10.3390/microorganisms10010155 (PMC8782003; doi:10.3390/microorganisms10010155)
Supplement: Supplementary file 1 [file microorganisms-10-00155-s001.zip › microorganisms-1521589-supplementary.pdf]

microorganisms

Supplementary Materials

Table S1. Distribution of the samples analysed by kind and light-sensitive layer material in single archives.

Archives	Depositars	Samples total	CIN[1]	PHNEG[1]	PHPOS[1]				
			GEL	GEL	all	GEL	ALB	COL	OTH
A	3	17	3	2	12	6	3	3	0
B	3	18	3	1	14	9	2	3	0
C	7	22	8	3	11	7	2	1	1[2]
D1	10	13	1	2	10	5	2	3	0
F	1	10	1	3	6	2	3	1	0
G2	4	7	1	2	4	1	1	1	1[2]
E	3	15	0	6	9	2	1	1	5[2,3,4,5,6]
D2	1	8	0	0	8	2	3	3	0
G1	1	3	0	0	3	2	0	1	0
G3	9	13	0	0	13	7	4	0	2[7,8]
Sum	42	126	17	19	90	43	21	17	9
% of all samples			13	15	71	34	17	13	7
% of PHPOS samples						48	23	19	10

[1] CIN-cinematographic material, PHNEG-photographic negative, PHPOS – photographic positive, GEL – gelatin, ALB – albumen, COL – collodion, OTH – other minor kinds, [2] – platinotype (platinum print), [3] – salt paper, [4] – gumoil printing,
[5] – cyanotype, [6] – ozotype, [7] – autotype, [8] - collotype.

Table S2. Distribution of analysed samples by the carrier and the light-sensitive layer material in single archives.

Archives	CIN[1]		PHNEG[1]				PHPOS[1]					
	GEL		GEL				GEL		ALB	COL		OTH
	CA	PES	CA	PES	CN	GL	BP	PAP	PAP	BP	PAP	PAP
A	2	1	0	0	0	2	6	0	3	3	0	0
B	3	0	1	0	0	0	9	0	2	3	0	0
C	7	1	0	0	2	1	7	0	2	1	0	1
D1	1	0	1	0	0	1	0	5	2	1	2	0
F	1	0	0	0	1	2	0	2	3	1	0	0
G2	1	0	0	0	1	1	1	0	1	1	0	1
E	0	0	2	1	1	2	2	0	1	1	0	5
D2	0	0	0	0	0	0	0	2	3	0	3	0
G1	0	0	0	0	0	0	2	0	0	1	0	0
G3	0	0	0	0	0	0	7	0	4	0	0	2
Sum	15	2	4	1	5	9	34	9	21	12	5	9
% of all samples	12	2	3	1	4	7	27	7	17	10	4	7
% of group samples[2]	88	12	21	5	26	47	79	21	100	71	29	100
% of PHPOS samples							38	10	23	13	6	10

[1] CIN-cinematographic material, PHNEG-photographic negative, PHPOS – photographic positive, GEL – gelatin, ALB – albumen, COL – collodion, OTH – other minor kinds (such as autotype, ozotype, collotype, cyanotype, platinotype, salt paper and gumoil printing), CA – cellulose acetate, PES – polyester, CN – cellulose nitrate, PAP – paper, BP – baryta paper, GL – glass, [2] group samples – by the kind and the light-sensitive layer.

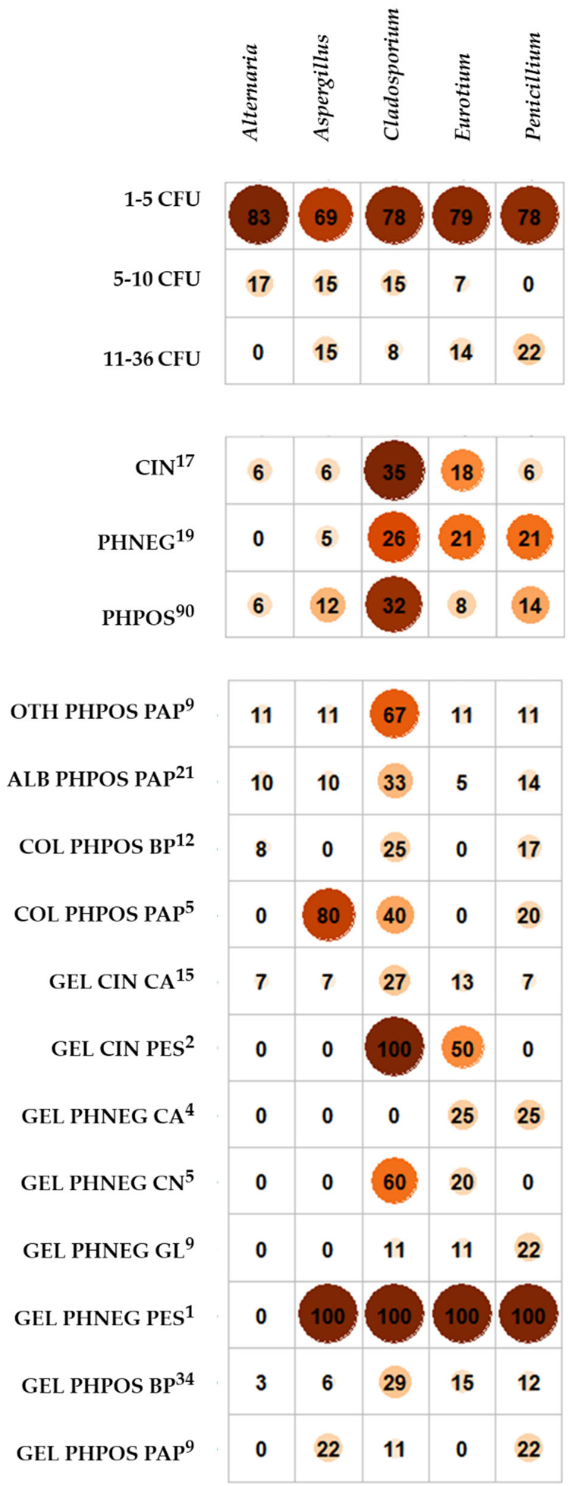

Figure S1. The relative frequencies of samples contaminated by five most frequent fungal genera due to different factors such as the number of CFU isolated from a sample, its isolation from single kinds of material (CIN, PHNEG, PHPOS), and the combination of the sample kind and material of light-sensitive layer and carrier. Legend: CIN-cinematographic material, PHNEG – photographic negative, PHPOS – photographic positive, GEL – gelatin, ALB – albumen, COL – collodion, OTH – other minor kinds (as autotype, ozotype, collotype, cyanotype, platinotype, salt paper and gumoil printing), CA – cellulose acetate, PES – polyester, CN – cellulose nitrate, PAP – paper, BP – baryta paper, GL – glass. The upper index indicates the number of analysed samples.

	Arthrobacter	*Bacillus*	*Dermacoccus*	*Kocuria*	*Micrococcus*	*Neisseria*	*Paenibacillus*	*Rothia*	*Staphylococcus*	*Streptococcus*
1-10 CFU	100	28	100	55	95	100	67	100	91	58
11-30 CFU	0	36	0	18	5	0	0	0	4	36
> 30 CFU	0	36	0	27	0	0	33	0	4	6
CIN^{17}	0	6	6	6	12	6	6	0	47	12
PHNEG^{19}	11	5	5	5	11	11	0	5	32	16
PHPOS^{90}	1	26	4	10	17	2	4	4	28	19
OTH PHPOS PAP^{9}	11	11	0	0	0	0	0	0	33	11
ALB PHPOS PAP^{21}	0	29	0	10	14	0	0	14	33	33
COL PHPOS BP^{12}	0	8	8	0	33	8	0	8	33	25
COL PHPOS PAP^{5}	0	20	0	20	0	0	20	0	40	0
GEL CIN CA^{15}	0	7	7	7	13	7	7	0	47	13
GEL CIN PES^{2}	0	0	0	0	0	0	0	0	50	0
GEL PHNEG CA^{4}	25	25	0	25	50	0	0	0	50	0
GEL PHNEG CN^{5}	0	0	0	0	0	20	0	0	0	20
GEL PHNEG GL^{9}	11	0	11	0	0	11	0	11	33	22
GEL PHNEG PES^{1}	0	0	0	0	0	0	0	0	100	0
GEL PHPOS BP^{34}	0	32	9	15	21	3	9	0	15	15
GEL PHPOS PAP^{9}	0	33	0	11	11	0	0	0	44	11

Figure S2. The relative frequencies of samples contaminated by ten most frequent bacterial genera due to different factors such as the number of CFU isolated from a sample (regarding single species), its isolation from single kinds of material (CIN, PHNEG, PHPOS), and the combination of the sample kind and material of light-sensitive layer and carrier. Legend: CIN-cinematographic material, PHNEG – photographic negative, PHPOS – photographic positive, GEL – gelatin, ALB – albumen, COL – collodion, OTH – other minor kinds (as autotype, ozotype, collotype, cyanotype, platinotype, salt paper and gumoil printing), CA – cellulose acetate, PES – polyester, CN – cellulose nitrate, PAP – paper, BP – baryta paper, GL – glass. The upper index indicates the number of analysed samples.
